# Supplementary material for: Transcriptional downregulation of MHC class I and melanoma de- differentiation in resistance to PD-1 inhibition
Source: Nat Commun. 2020 Apr 20;11:1897. doi: 10.1038/s41467-020-15726-7 (PMC7171183; doi:10.1038/s41467-020-15726-7)
Supplement: Supplementary file 1 — Supplementary Information [file 41467_2020_15726_MOESM1_ESM.pdf]

Transcriptional downregulation of MHC class I and melanoma de-differentiation in resistance to PD-1 inhibition

Lee JH et al.

Figure S1

A

Riaz *et al.* 2017
(anti-PD-1)

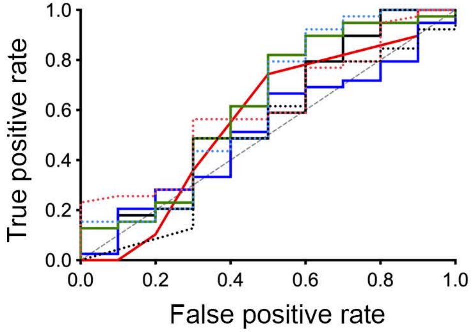

Signature	AUC	p
IPRES	0.5179	0.8621
IMPRES	0.5667	0.5189
CYT score	0.6205	0.2436
TIDE	0.5769	0.4568
18-immune gene set	0.6128	0.2751
CD8A/CSF1R ratio	0.6026	0.3211
CD8+ T cells CIBERSORT	0.5141	0.8915

Hugo *et al.* 2016
(anti-PD-1)

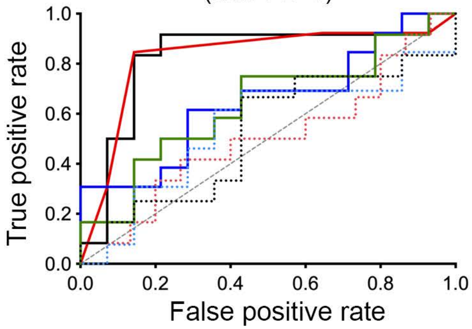

Signature	AUC	p
IPRES	0.6190	0.3036
IMPRES	0.8214	0.0055
CYT score	0.6369	0.2368
TIDE	0.8274	0.0047
18-immune gene set	0.5774	0.5037
CD8A/CSF1R ratio	0.5056	0.9611
CD8+ T cells CIBERSORT	0.5238	0.8370

Van Allen *et al.* 2015
(anti-CTLA4)

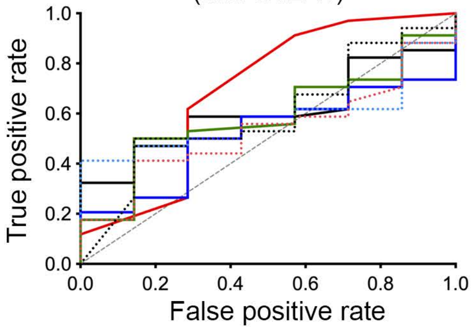

Signature	AUC	p	AUC[1]	p[1]
IPRES	0.5168	0.8898	0.6659	0.0669
IMPRES	0.6891	0.1190	0.7632	0.0037
CYT score	0.5882	0.4669	0.6911	0.0349
TIDE	0.6071	0.3770	0.8021	0.0009
18-immune gene set	0.5798	0.5103	0.7277	0.0119
CD8A/CSF1R ratio	0.5336	0.7816	0.6499	0.0979
CD8+ T cells CIBERSORT	0.5945	0.4356	0.7101	0.0223

[1]Performance of signatures in patients who showed clinical benefit (CR, PR or SD with overall survival >1 year) vs no clinical benefit (PD or SD with overall survival <1 year), as defined in the original study

Supplementary Figure 1 Performance of immune-predictive transcriptome scores

Immune-predictive transcriptome scores derived for three publicly available immune checkpoint inhibitor melanoma datasets (GSE78220, GSE91061 and TPM-RSEM values for the Van Allen dataset[1] from GitHub: https://github.com/vanallenlab/VanAllen_CTLA4_Science_RNASeq_TPM. PRE-treatment melanoma biopsies and patient response data (CR/PR vs SD/PD) were used to generate receiver operator characteristic (ROC) curves measuring the performance of each indicated signature in predicting PD-1 inhibitor responses in 49 patients treated with the PD-1 inhibitor, nivolumab[2], 26 patients treated PD-1 inhibitors nivolumab or pembrolizumab[3] and 41 patients treated with anti-CTLA4 with RECIST data[1]. We also examined the transcriptome signatures in patients treated with anti-CTLA4 stratified according to clinical benefit vs no-clinical benefit as defined in the original report. Clinical benefit was defined as CR, PR or SD by RECIST with OS greater than 1 year and no clinical benefit was defined as PD of SD with OS less than 1 year[2]. The resulting AUCs and p values are tabulated. The signatures applied to our dataset were derived from the following references: IPRES signature[3], IMPRES signature[4], *CD8A/CSF1R* ratio[5], 18-immune gene set[6], TIDE[7], CYT score[8] and CIBERSORT estimated relative proportion of CD8+ T cells[9].

Figure S2

A

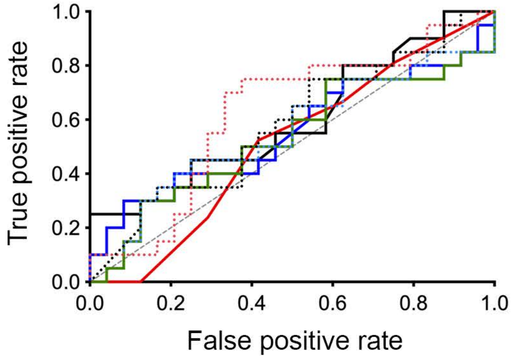

Signature	AUC	p
IPRES	0.5542	0.5400
IMPRES	0.5099	0.9094
CYT score	0.5313	0.7237
TIDE	0.5979	0.2679
18-immune gene set	0.5563	0.5245
CD8A/CSF1R ratio	0.6229	0.1643
CD8+ T cells CIBERSORT	0.5813	0.3580

B

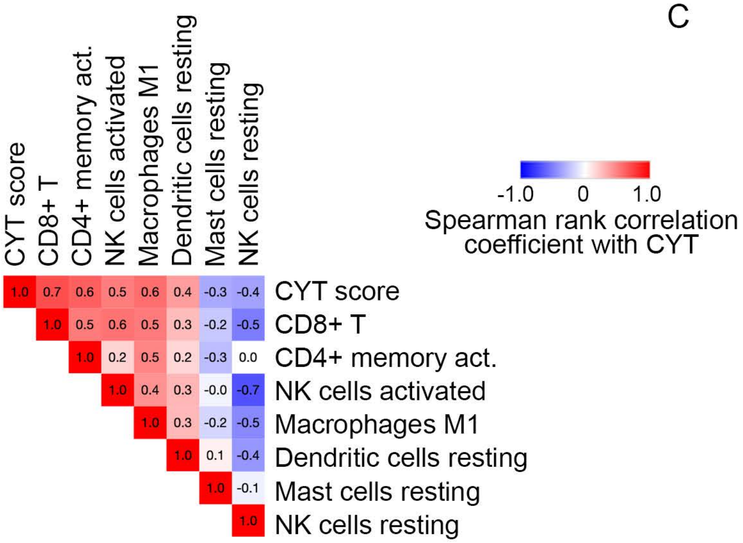

C

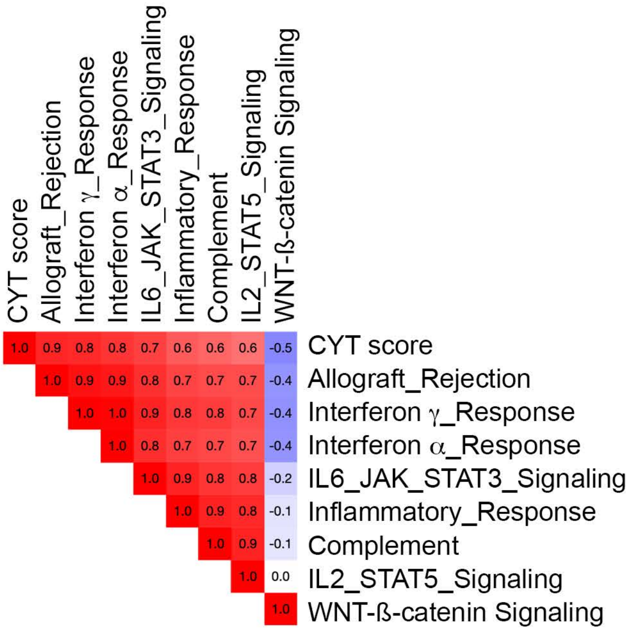

D

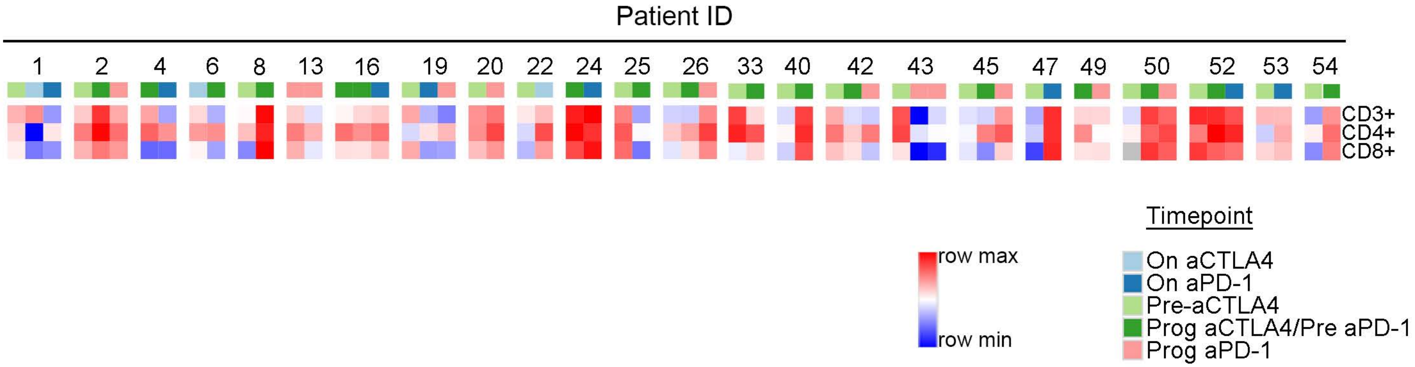

Supplementary Figure 2 Immune profiling in melanoma biopsies

(A) Immune-predictive transcriptome scores derived for each PRE-treatment melanoma biopsy (n=44) and patient or lesion response data were used to generate receiver operator characteristic (ROC) curves measuring the performance of each indicated signature in predicting PD-1 inhibitor responses in our patient cohort. In these analyses the response of four patients was changed to reflect the lesion-specific response assessment of the pre-treatment tumor (see Table 2). The resulting AUCs and p values are tabulated. The signatures applied to our dataset were derived from the following references: IPRES signature[3], IMPRES signature[4], *CD8A/CSF1R* ratio[5], 18-immune gene set[6], TIDE[7], CYT score[8] and CIBERSORT estimated relative proportion of CD8+ T cells[9]

(B) Correlation matrix of intra-tumoral cytolytic activity score (CYT, expression of *PRF1* and GZMA[8]) with CIBERSORT immune cell subset scores[9] in 79 melanoma biopsies. The Spearman rank correlation coefficients are shown within the matrix, and the false discovery adjusted p-value was < 0.01 for all signatures shown (see Supplementary Data 7)

(C) Correlation matrix of intra-tumoral cytolytic activity score (CYT, expression of *PRF1* and GZMA[8]) with ssGSEA score derived from the indicated Hallmark immune-related signatures. The Spearman rank correlation coefficients are shown within the matrix, and the false discovery adjusted p-value was < 0.01 for all signatures shown (see Supplementary Data 7)

(D) Heatmap showing immune cell profiling by IHC in longitudinal melanoma biopsies (pre-treatment, early on-treatment and late on-treatment) in patients undergoing sequential treatment with PD-1 or CTLA-4 inhibitors. Data derived from[10]

Figure S3

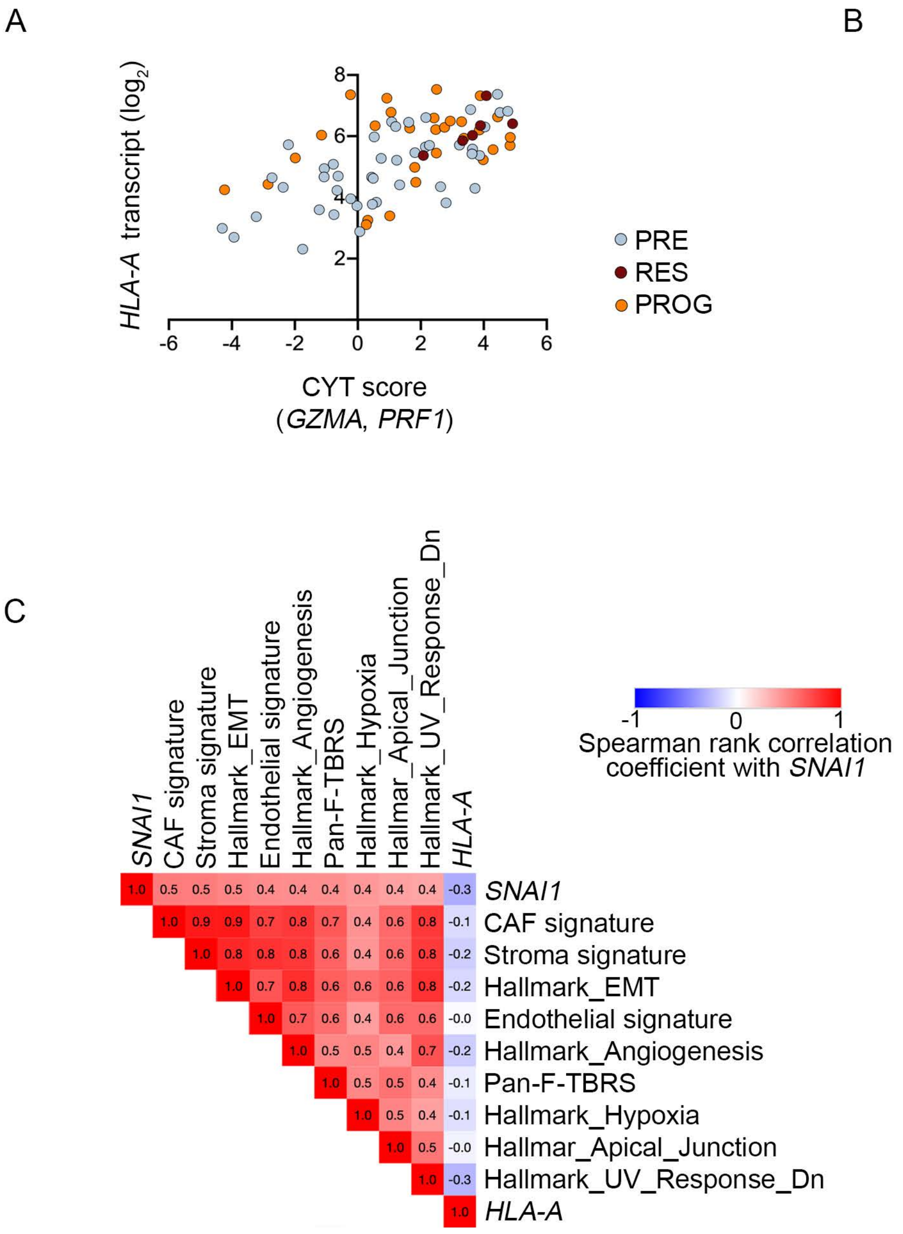
A
HLA-A transcript (log2)
8
6
4
2
-6
-4
-2
0
2
4
6
CYT score
(GZMA, PRF1)
PRE
RES
PROG
B
CYT score
(GZMA, PRF1)
6
4
2
0
-2
-4
HLA-A
high
HLA-A
low
C
SNAI1
CAF signature
Stroma signature
Hallmark_EMT
Endothelial signature
Hallmark_Angiogenesis
Pan-F-TBRS
Hallmark_Hypoxia
Hallmar_Apical_Junction
Hallmark_UV_Response_Dn
HLA-A
-1
0
1
Spearman rank correlation
coefficient with SNAI1
1.0 0.5 0.5 0.5 0.4 0.4 0.4 0.4 0.4 0.4 -0.3 SNAI1
1.0 0.9 0.9 0.7 0.8 0.7 0.4 0.6 0.8 -0.1 CAF signature
1.0 0.8 0.8 0.8 0.6 0.4 0.6 0.8 -0.2 Stroma signature
1.0 0.7 0.8 0.6 0.6 0.6 0.8 -0.2 Hallmark_EMT
1.0 0.7 0.6 0.4 0.6 0.6 -0.0 Endothelial signature
1.0 0.5 0.5 0.4 0.7 -0.2 Hallmark_Angiogenesis
1.0 0.5 0.5 0.4 -0.1 Pan-F-TBRS
1.0 0.5 0.4 -0.1 Hallmark_Hypoxia
1.0 0.5 -0.0 Hallmar_Apical_Junction
1.0 -0.3 Hallmark_UV_Response_Dn
1.0 HLA-A

Supplementary Figure 3 Association of transcriptome signatures with *HLA-A* downregulation and *SNAI1* transcript expression

(A) Scatter plot showing the relationship between CYT score and the expression of the *HLA-A* transcript in responding (RES; n=6), pre-treatment (PRE; n=44) and progressing biopsies (PROG; n=29).

(B) Plots showing CYT score in the selected CYT score-matched tumors (n = 38) with high or low *HLA-A* transcript expression.

(C) Correlation matrix of *SNAI1* gene expression with ssGSEA scores derived from the Hallmark gene set collection and stromal cell-specific transcriptome signatures [11] in the TCGA SKCM dataset. The Spearman rank correlation coefficients are shown within the matrix, and the false discovery adjusted p-value was < 0.01 for all signatures shown (see also Supplementary Data 7).

Figure S4

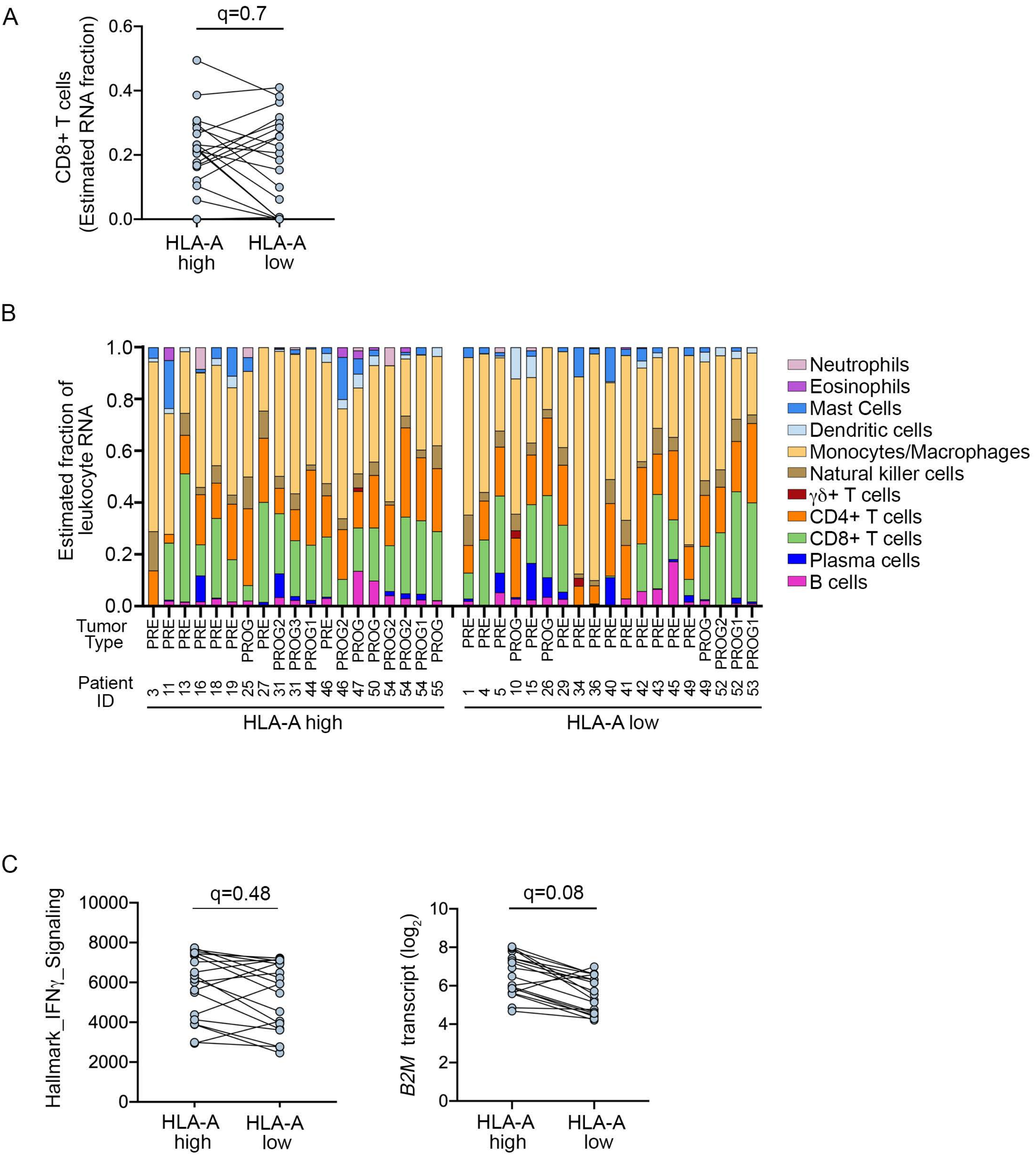
A
q=0.7
CD8+ T cells (Estimated RNA fraction)
0.6
0.4
0.2
0.0
HLA-A high
HLA-A low
B
Estimated fraction of leukocyte RNA
1.0
0.8
0.6
0.4
0.2
0.0
Neutrophils
Eosinophils
Mast Cells
Dendritic cells
Monocytes/Macrophages
Natural killer cells
γδ+ T cells
CD4+ T cells
CD8+ T cells
Plasma cells
B cells
Tumor Type
Patient ID
HLA-A high
HLA-A low
C
q=0.48
Hallmark_IFNγ_Signaling
10000
8000
6000
4000
2000
0
HLA-A high
HLA-A low
q=0.08
B2M transcript (log2)
10
8
6
4
2
0
HLA-A high
HLA-A low

Supplementary Figure 4 *HLA-A* transcript downregulation and immune cell subset and activation signatures

(A) Plot showing CD8+ T cell estimated fraction (CIBERSORT relative score) in the CYT score-matched tumors (n = 38) with low or high *HLA-A* transcript expression. FDR-adjusted p values (q) calculated using limma test.

(B) Fractions of 11 major leukocyte cell subsets called by CIBERSORT in CYT score matched tumors (n=38) with high and low *HLA-A* transcript. No significant differences were observed in any immune subsets in the *HLA-A* high expression vs *HLA-A* low expression tumor subgroups (see Supplementary Data 6).

(C) Plots showing Hallmark_IFNγ_signaling (ssGSEA scores) and transcript levels *B2M* in the CYT score-matched tumors (n = 38) with low or high *HLA-A* transcript expression. FDR-adjusted p values (q) calculated using limma test.

Figure S5

A

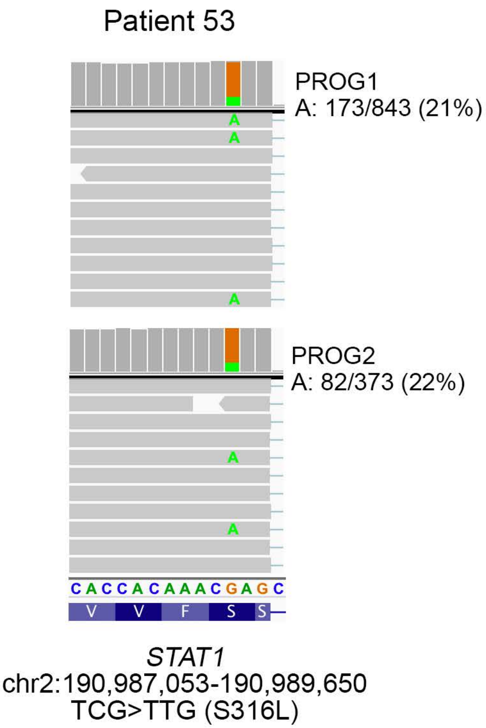
Patient 53
PROG1
A: 173/843 (21%)
PROG2
A: 82/373 (22%)
C A C C A C A A A C G A G C
V V F S S
STAT1
chr2:190,987,053-190,989,650
TCG>TTG (S316L)

B

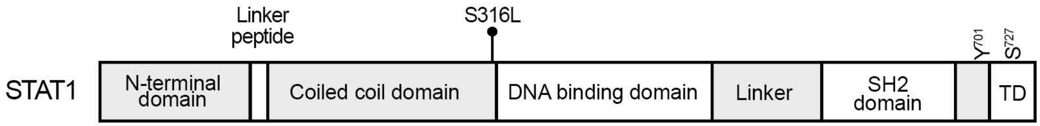
Linker peptide
S316L
Y701
S727
STAT1
N-terminal domain
Coiled coil domain
DNA binding domain
Linker
SH2 domain
TD

Supplementary Figure 5 STAT1 missense mutations in PD-1 PROG tumors

(A) STAT1 missense mutation identified in the PD-1 PROG tumors derived from patient 53. The IGV compressed window shows wild type and variant alleles with variant frequency shown.

(B) Schematic STAT1 protein showing functional domains and activating phosphorylation sites and the S316L mutation identified in Patient 53. TD, transactivation domain.

Figure S6

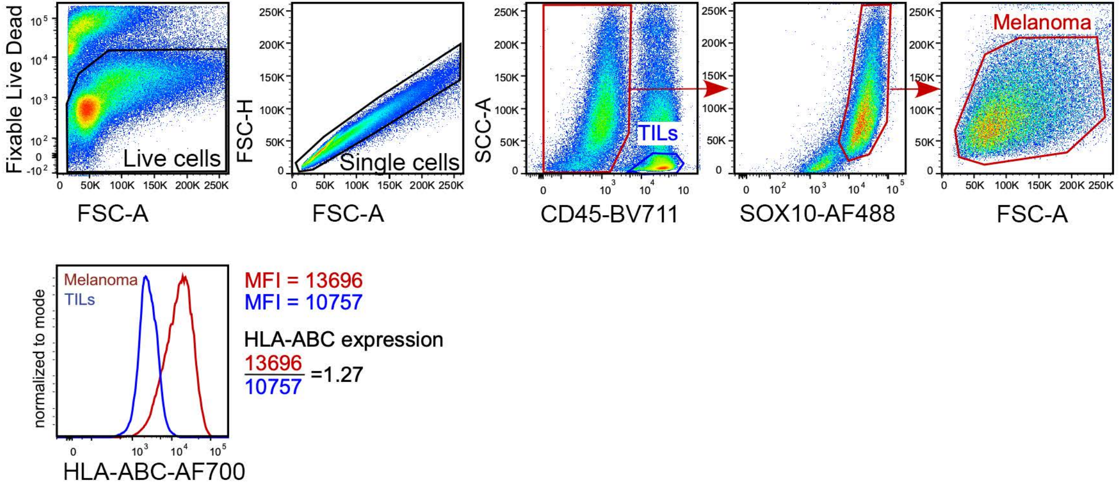
Fixable Live Dead
Live cells
FSC-A
FSC-H
Single cells
FSC-A
SCC-A
TILs
CD45-BV711
SOX10-AF488
Melanoma
FSC-A
Melanoma
TILs
normalized to mode
HLA-ABC-AF700
MFI = 13696
MFI = 10757
HLA-ABC expression
13696 / 10757 =1.27

Supplementary Figure 6 Analysis of HLA-ABC expression in enzymatically dissociated tumors

Dissociated tumors were gated for live cells and single cells; melanoma cells were identified as CD45-negative, SOX10-positive, forward and side scatter-high events (red), while tumor infiltrating lymphocytes (TILs) were identified as CD45-positive, side scatter-low events (blue).

Relative HLA-ABC expression (bottom panel) was determined as a ratio of geometric mean fluorescence intensities (MFI) of melanoma cells and TILs within each sample.

Figure S7

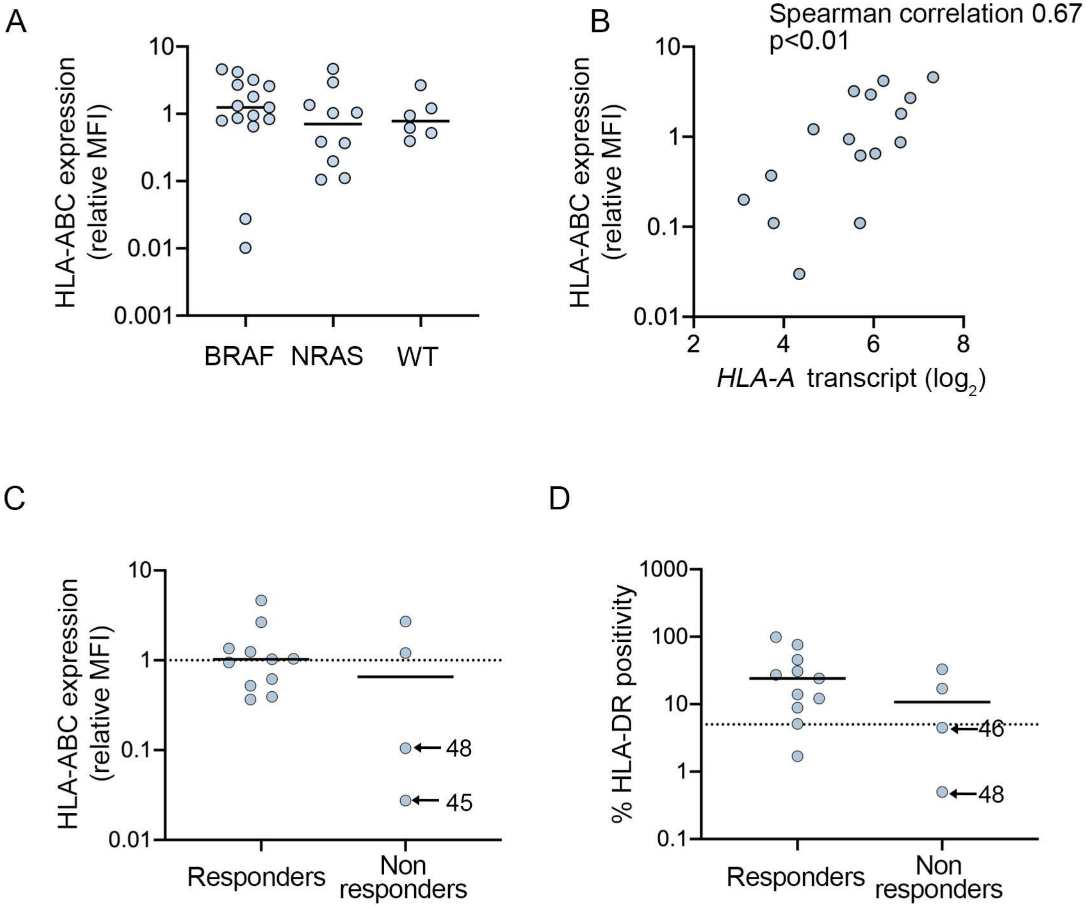

A
HLA-ABC expression (relative MFI)
10
1
0.1
0.01
0.001
BRAF
NRAS
WT
B
Spearman correlation 0.67
p<0.01
HLA-ABC expression (relative MFI)
10
1
0.1
0.01
2
4
6
8
HLA-A transcript (log_2)
C
HLA-ABC expression (relative MFI)
10
1
0.1
0.01
48
45
Responders
Non responders
D
% HLA-DR positivity
1000
100
10
1
0.1
46
48
Responders
Non responders

Supplementary Figure 7 Analysis HLA-ABC expression in BRAF-mutant, RAS-mutant and BRAF/RAS wild type melanoma

(A) Cell surface expression of HLA-ABC in 31 melanoma tumors with defined oncogenic driver mutations, including 15 *BRAF*-mutant, 10 *N/KRAS*-mutant and 6 *BRAF/NRAS* wild type (WT). HLA-ABC expression is calculated relative to HLA-ABC in tumor-infiltrating lymphocytes. There was no significant difference in the relative HLA-ABC expression between three melanoma genotypes; Kruskal-Wallis test with Dunn's multiple comparison test.

(B) Scatter plot showing the relationship between *HLA-A* transcript expression and HLA-ABC cell surface expression score in 16 melanoma tumors with flow cytometry and RNA sequence data.

(C) Cell surface expression of HLA-ABC (relative to HLA-ABC in tumor-infiltrating lymphocytes) in melanoma cells derived from fresh dissociates of pre-treatment tumors grouped according to patient response to PD-1 inhibition. Solid lines represent median and dotted line set at Y=1. Patients with CR or PR were classified as responders, while patients with SD and PD were classified as non-responders

(D) Cell surface expression of HLA-DR (percent HLA-DR positivity) in melanoma cells derived from fresh dissociates of pre-treatment tumors grouped according to patient response to PD-1 inhibition. Solid lines represent median and dotted line set at Y=5%. Patients with CR or PR were classified as responders, while patients with SD and PD were classified as non-responders.

Figure S8

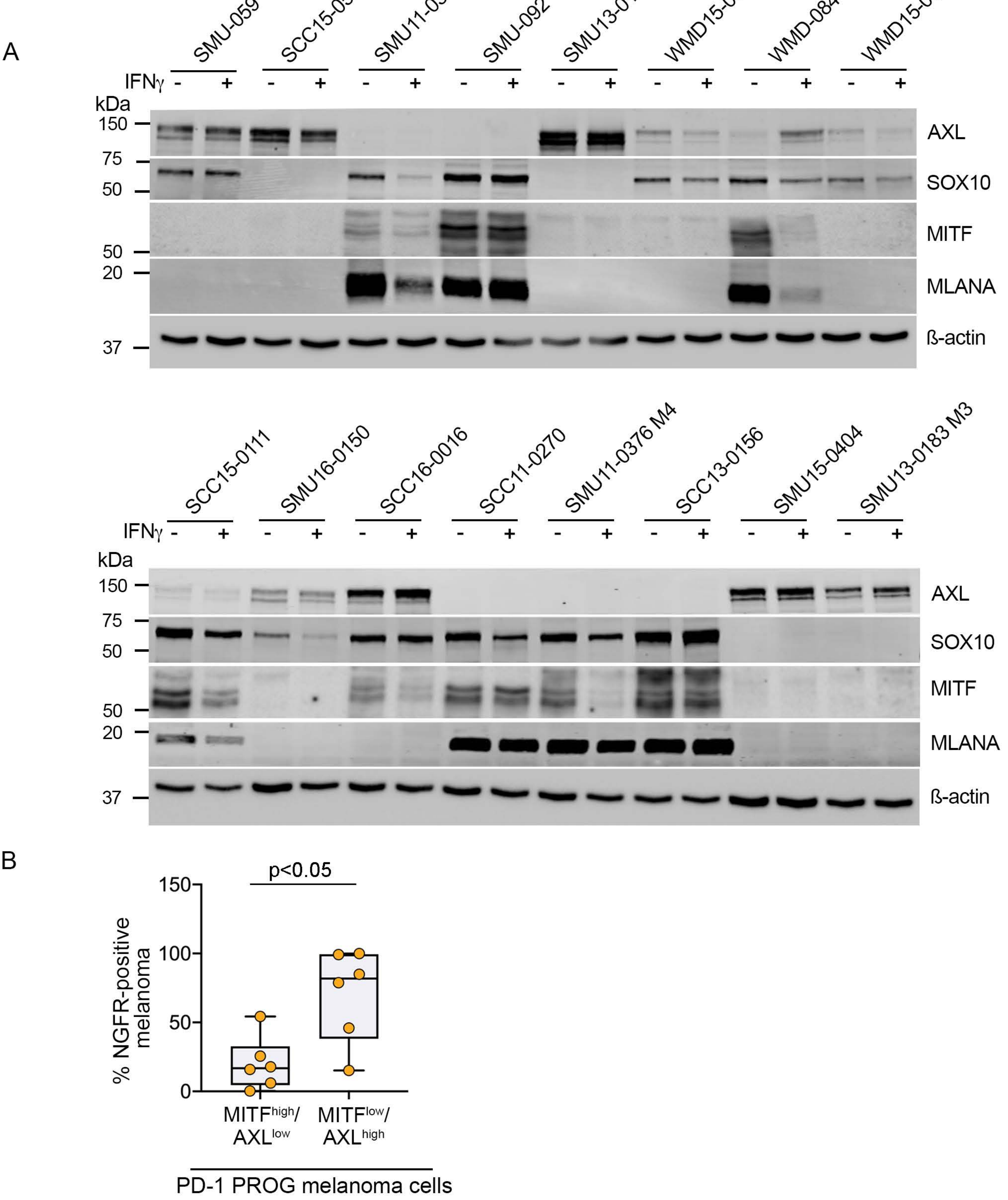
A
SMU-059
SCC15-0534
SMU11-0376 M2
SMU-092
SMU13-0183 M7
WMD15-083 #1
WMD-084
WMD15-083 #2
IFNγ - + - + - + - + - + - + - + - +
kDa
150
75
50
50
20
37
AXL
SOX10
MITF
MLANA
ß-actin
SCC15-0111
SMU16-0150
SCC16-0016
SCC11-0270
SMU11-0376 M4
SCC13-0156
SMU15-0404
SMU13-0183 M3
IFNγ - + - + - + - + - + - + - + - +
kDa
150
75
50
50
20
37
AXL
SOX10
MITF
MLANA
ß-actin
B
p<0.05
150
100
50
0
% NGFR-positive melanoma
MITF^{high}/ AXL^{low}
MITF^{low}/ AXL^{high}
PD-1 PROG melanoma cells

Supplementary Figure 8 MITF^{low}/AXL^{high} de-differentiation in short term PD-1 PROG cell lines

(A) Western blots of cell lysates showing protein markers of differentiation (SOX10, MLANA, MITF) and de-differentiation (AXL) 24 h after treating cells with vehicle (-) or 1000 U/ml IFNγ (+). REVERT stain shown in Supplementary Figure 11B.

(B) Percentage of NGFR-positive (de-differentiated) melanoma cells in fresh tumor samples used to derive matching short-term PD-1 PROG melanoma cell lines with the MITF^{high}/AXL^{low} (differentiated phenotype) or MITF^{low}/AXL^{high} dedifferentiated phenotype. Box plots show the median and interquartile ranges, and data were compared using Mann Whitney test.

Figure S9

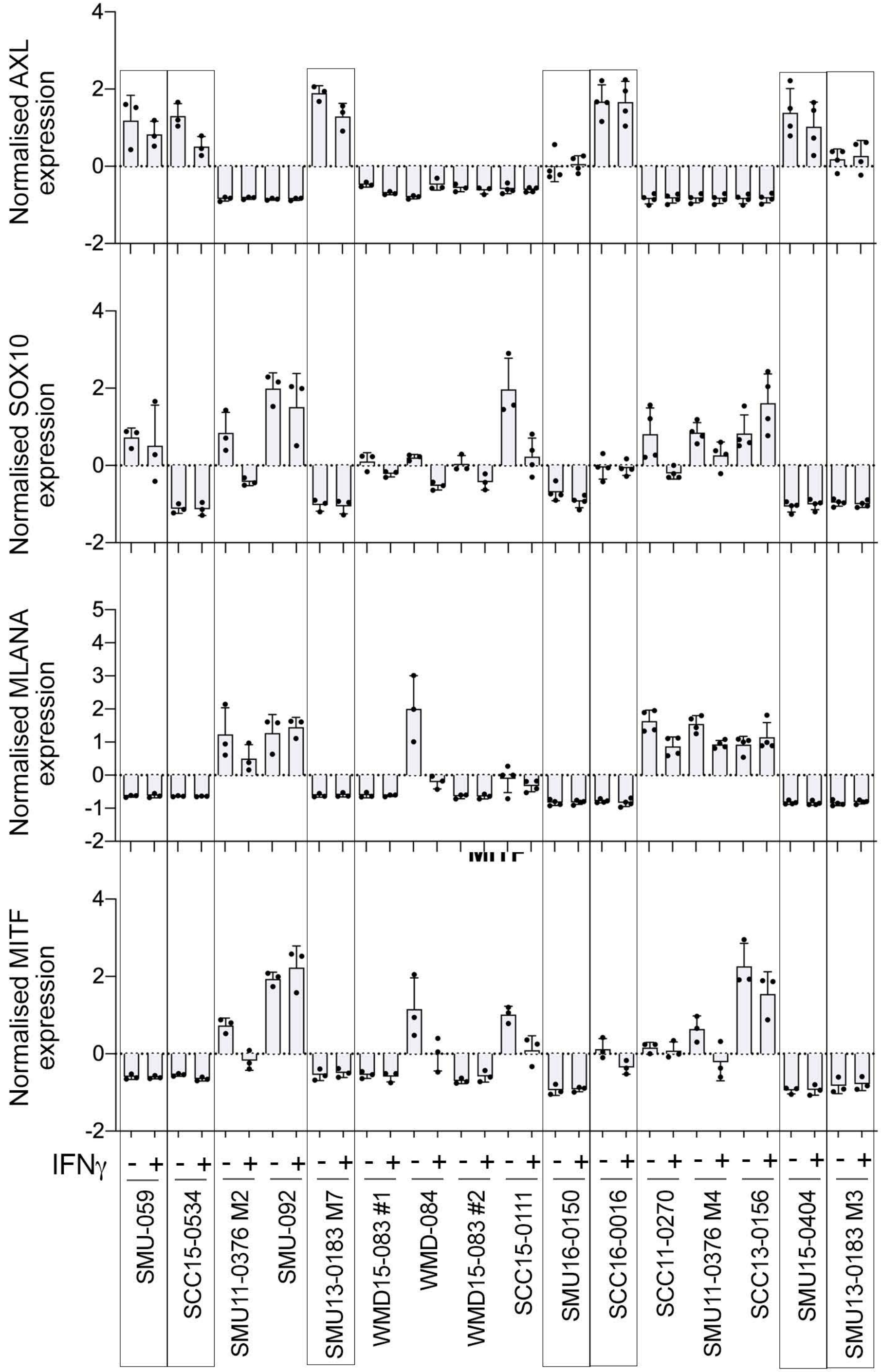
Normalised AXL expression
Normalised SOX10 expression
Normalised MLANA expression
Normalised MITF expression
IFNγ
SMU-059
SCC15-0534
SMU11-0376 M2
SMU-092
SMU13-0183 M7
WMD15-083 #1
WMD-084
WMD15-083 #2
SCC15-0111
SMU16-0150
SCC16-0016
SCC11-0270
SMU11-0376 M4
SCC13-0156
SMU15-0404
SMU13-0183 M3

Supplementary Figure 9 Quantitation of melanoma de-differentiation protein markers in PD-1 PROG cell lines

Quantitation of AXL, SOX10, MLANA expression normalised to REVERT and MITF expression normalised to ß-actin (converted to z-scores to enable analysis of three biological western blot replicates). Average and standard deviation of z-scores are shown and representative western blot images are shown in Supplementary Figure 8. Melanoma cell lines classified as de-differentiated based on $MITF^{low}/AXL^{high}$ expression are boxed.

Figure S10

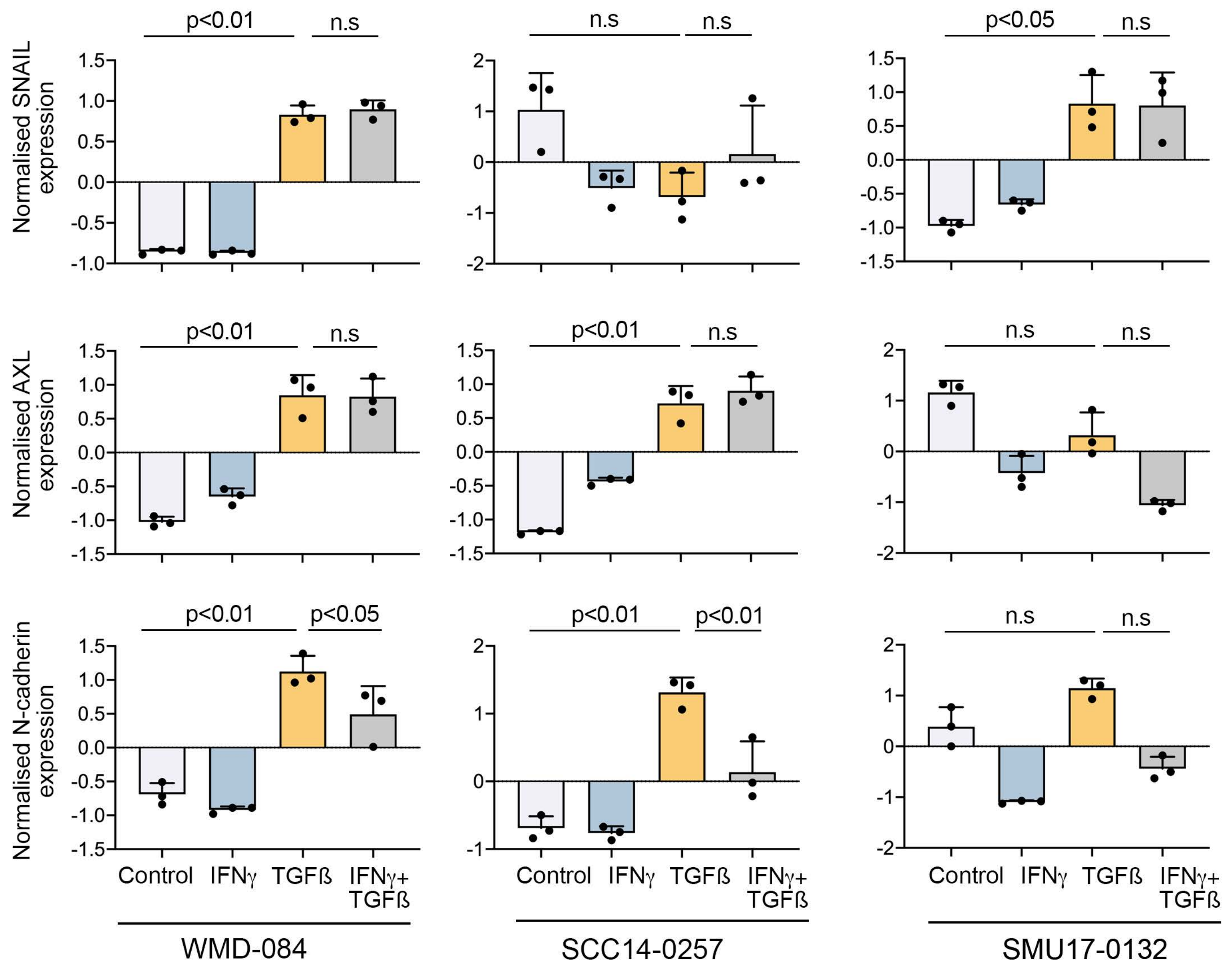

p<0.01
n.s
p<0.05
p<0.01
p<0.05
Normalised SNAIL expression
Normalised AXL expression
Normalised N-cadherin expression
Control
IFNγ
TGFß
IFNγ+ TGFß
WMD-084
SCC14-0257
SMU17-0132

Supplementary Figure 10 Quantitation of melanoma de-differentiation protein markers in response to IFNγ and/or TGFß in melanoma cell lines

Quantitation of de-differentiation markers SNAIL, AXL and N-cadherin normalised to REVERT stain in WMD-084, SCC14-0257 and SMU17-0132 melanoma cells treated with vehicle (Control), 1000 U/ml IFNγ- and/or 10 ng/ml TGFß for 72 h. Normalised protein expression data were converted to z-scores to enable analysis of three biological western blot replicates. Average and standard deviation of z-scores are shown and representative western blot images are shown in Figure 5C. Data were compared using one-way ANOVA with the Geisser-Greenhouse correction.

References

1. Van Allen, E.M., *et al.* Genomic correlates of response to CTLA4 blockade in metastatic melanoma. *Science* (2015).
2. Riaz, N., *et al.* Tumor and Microenvironment Evolution during Immunotherapy with Nivolumab. *Cell* **171**, 934-949.e915 (2017).
3. Hugo, W., *et al.* Genomic and Transcriptomic Features of Response to Anti-PD-1 Therapy in Metastatic Melanoma. *Cell* **165**, 35-44 (2016).
4. Auslander, N., *et al.* Robust prediction of response to immune checkpoint blockade therapy in metastatic melanoma. *Nat Med* **24**, 1545-1549 (2018).
5. Neubert, N.J., *et al.* T cell-induced CSF1 promotes melanoma resistance to PD1 blockade. *Sci Transl Med* **10**(2018).
6. Ayers, M., *et al.* IFN-gamma-related mRNA profile predicts clinical response to PD-1 blockade. *J Clin Invest* **127**, 2930-2940 (2017).
7. Jiang, P., *et al.* Signatures of T cell dysfunction and exclusion predict cancer immunotherapy response. *Nat Med* (2018).
8. Rooney, M.S., Shukla, S.A., Wu, C.J., Getz, G. & Hacohen, N. Molecular and genetic properties of tumors associated with local immune cytolytic activity. *Cell* **160**, 48-61 (2015).
9. Newman, A.M., *et al.* Robust enumeration of cell subsets from tissue expression profiles. *Nat Methods* **12**, 453-457 (2015).
10. Chen, P.L., *et al.* Analysis of Immune Signatures in Longitudinal Tumor Samples Yields Insight into Biomarkers of Response and Mechanisms of Resistance to Immune Checkpoint Blockade. *Cancer Discov* **6**, 827-837 (2016).
11. Jerby-Arnon, L., *et al.* A Cancer Cell Program Promotes T Cell Exclusion and Resistance to Checkpoint Blockade. *Cell* **175**, 984-997.e924 (2018).
