# Supplementary material for: Transcriptional downregulation of MHC class I and melanoma de- differentiation in resistance to PD-1 inhibition
Source: Nat Commun. 2020 Apr 20;11:1897. doi: 10.1038/s41467-020-15726-7 (PMC7171183; doi:10.1038/s41467-020-15726-7)
Supplement: Supplementary file 3 — Description of Additional Supplementary Files [file 41467_2020_15726_MOESM3_ESM.docx]

**Description of Additional Supplementary Files**

**File name: Supplementary Data 1**

**Description: Complete list of 68 melanoma patients used in this study with treatment details, patient and tumor outcomes.**

**File name: Supplementary Data 2**

**Description: Individual tumor scores for seven immunotherapy predictive signatures.**

**File name: Supplementary Data 3**

**Description: Mutations identified in genes related to antigen presentation and IFNγ signalling.**

**File name: Supplementary Data 4**

**Description: Genes differentially expressed (q<0.01) in CYT-matched melanoma tumours with low vs high HLA-A transcript expression.**

**File name: Supplementary Data 5**

**Description: Gene-sets differentially expressed (q<0.01) in CYT-matched melanoma tumours with low vs high HLA-A transcript expression.**

**File name: Supplementary Data 6**

**Description: Differential immune cell abundance based on CIBERSORT relative estimates in CYT-matched melanoma tumours with low vs high HLA-A transcript expression.**

**File name: Supplementary Data 7**

**Description: Spearman correlation data for Figures 3D, S2B and S2C**
